# Mass Trapping and Larval Source Management for Mosquito Elimination on Small Maldivian Islands

**DOI:** 10.3390/insects13090805

**Published:** 2022-09-02

**Authors:** Akib Jahir, Najat F. Kahamba, Tom O. Knols, Gordon Jackson, Nila F. A. Patty, Sonu Shivdasani, Fredros O. Okumu, Bart G. J. Knols

**Affiliations:** 1 Culex Maldives, 4th Floor Jazeera Building, Boduthakurufaanu Magu, Male 20077, Maldives; 2 Soneva Fushi, 4th Floor Jazeera Building, Boduthakurufaanu Magu, Male 20077, Maldives; 3 Ifakara Health Institute, Ifakara P.O. Box 53, Tanzania; 4 K&S Holding BV, Kalkestraat 20, 6669 CP Dodewaard, The Netherlands

**Keywords:** mosquito, elimination, islands, trapping, larval source management, arbovirus

## Abstract

**Simple Summary:**

The globalization of trade and travel, in combination with climate change, have resulted in the geographical expansion of mosquito-borne diseases. Moreover, over-reliance on chemical pesticides to control mosquitoes has resulted in resistance, which threatens the management of disease risk. We show, for the first time, that mosquito traps baited with human odors, in combination with controlling mosquito larvae in breeding sites, resulted in the near elimination of mosquito populations on two small islands, and the elimination of *Aedes* mosquitoes for 6+ months on a third island, in the Maldives. The levels of control achieved are comparable to current genetic control methods that are far more costly and impractical for implementation on small islands. The approach presented here poses the first alternative in decades to manage mosquito-borne disease risk on small (tropical) islands in an affordable and environmentally friendly manner.

**Abstract:**

Globally, environmental impacts and insecticide resistance are forcing pest control organizations to adopt eco-friendly and insecticide-free alternatives to reduce the risk of mosquito-borne diseases, which affect millions of people, such as dengue, chikungunya or Zika virus. We used, for the first time, a combination of human odor-baited mosquito traps (at 6.0 traps/ha), oviposition traps (7.2 traps/ha) and larval source management (LSM) to practically eliminate populations of the Asian tiger mosquito *Aedes albopictus* (peak suppression 93.0% (95% CI 91.7–94.4)) and the Southern house mosquito *Culex quinquefasciatus* (peak suppression 98.3% (95% CI 97.0–99.5)) from a Maldivian island (size: 41.4 ha) within a year and thereafter observed a similar collapse of populations on a second island (size 49.0 ha; trap densities 4.1/ha and 8.2/ha for both trap types, respectively). On a third island (1.6 ha in size), we increased the human odor-baited trap density to 6.3/ha and then to 18.8/ha (combined with LSM but without oviposition traps), after which the *Aedes* mosquito population was eliminated within 2 months. Such suppression levels eliminate the risk of arboviral disease transmission for local communities and safeguard tourism, a vital economic resource for small island developing states. Terminating intense insecticide use (through fogging) benefits human and environmental health and restores insect biodiversity, coral reefs and marine life in these small and fragile island ecosystems. Moreover, trapping poses a convincing alternative to chemical control and reaches impact levels comparable to contemporary genetic control strategies. This can benefit numerous communities and provide livelihood options in small tropical islands around the world where mosquitoes pose both a nuisance and disease threat.

## 1. Introduction

Mosquito-borne arboviruses threaten a third of the world’s population and continue to expand due to human disturbances of natural ecosystems, the globalization of trade and travel and climate change [1]. In the absence of effective vaccines or specific drugs against many of these diseases, such as dengue, Chikungunya or Zika virus, chemical control of mosquitoes remains the most commonly deployed strategy to curb transmission. However, environmental impacts and widespread resistance to insecticides undermine the effectiveness thereof and call for new and more sustainable tools [2,3]. In recent years, the Sterile Insect Technique (SIT), based on genetic modification [4], or the use of *Wolbachia* bacteria to induce sterility in populations [5,6], have been piloted, with reductions in target mosquito populations exceeding 90%. However, besides being very expensive and requiring the production, sexing and release of millions of male mosquitoes over large areas, which is technically challenging at present, the straightforward replication of SIT in many countries and (remote) islands where it could be used remains logistically complex. Autodissemination traps, whereby the egg-laying female is contaminated with a larvicide (e.g., pyriproxyfen) that she carries to other breeding sites [7,8], have been developed but their effectiveness in terms of controlling disease transmission remains unknown. Although odor-baited traps for host-seeking mosquitoes have successfully been deployed to reduce malaria on an island in Lake Victoria, Kenya [9], no such trials have been undertaken in geographically isolated islands against the Asian tiger mosquito (*Aedes albopictus*, Skuse), a species capable of vectoring at least fourteen different arboviral diseases [10,11].

The Maldives archipelago, located southwest of India, falls within the native range of *Ae. albopictus*. It is the smallest Asian country by land area (296 km^2^), with a human population of circa half a million, and consists of 20 atolls and a total of 1192 coral islands, of which fewer than 200 are inhabited. Tourism (1.7 million visitors in 2019) provides a vital source of income, accounting for 28% of the GDP and more than 60% of foreign exchange receipts [12]. Although the country successfully eliminated malaria by 1984 [13] and lymphatic filariasis by 2007 [14], it sustains endemic dengue [15], chikungunya [16] and likely Zika virus [17]. The risk of dengue alone lowers the country’s gross annual income by USD 110 per resident and its annual tax receipts by USD 14 per resident [18].

**Figure 1:**
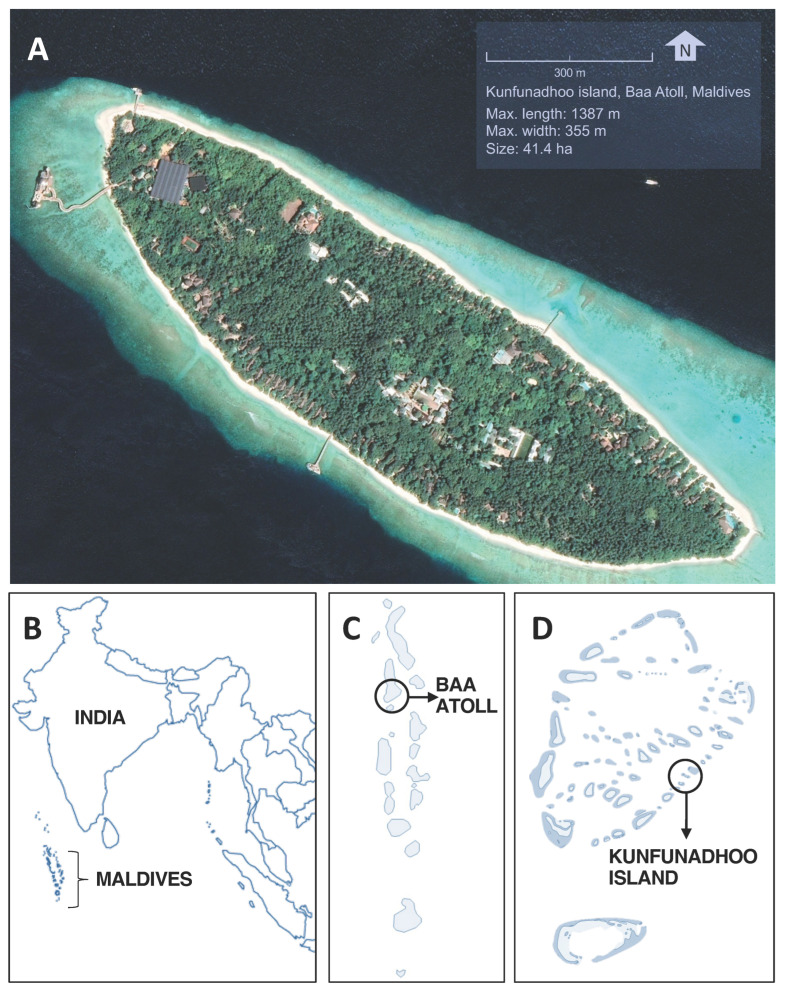
Location of Kunfunadhoo island. (**A**): Google Earth satellite image of Kunfunadhoo island (imagery date 27 December 2018). (**B**): Location of the Maldives. (**C**): Location of the Baa atoll. (**D**): Location of Kunfunadhoo island. The geographical center of the island is located at 5°06′42.81″ N, 73°04′40.31″ E.

Given the importance of tourism and the current problems with insecticide-based control methods, we pioneered an approach to drastically reduce mosquito populations on three small Maldivian islands. The approach was based on mass trapping and intense larval source management (LSM). Kunfunadhoo island, situated in the Baa atoll, comprises 41.4 hectares of dense tropical jungle (Figure 1, Supplementary Material Video S1), leased and occupied by Soneva Fushi (www.soneva.com, accessed on 1 September 2022), an award-winning high-end luxury villa resort. Some 420 staff live on the island, and, during peak occupancy periods, the total number of people on the island may reach 700 (Soneva, unpublished data). Both the Asian tiger mosquito and Southern house mosquito (*Culex quinquefasciatus*, Say) are present, the former often in high densities, whereas the latter occurs in lower numbers. For over two decades, the resort hired a professional pest control company that sprayed (misting or hot fogging; Supplementary Material Video S2) insecticides on a daily basis for most of the year to reduce nuisance biting and the risk of mosquito-borne disease transmission. At present, this insecticide-based approach remains the most common strategy across the Maldives, both in resort islands and local community islands. At Soneva, this third-party contract was terminated in March 2019 because of suspected resistance to the synthetic pyrethroid insecticides used, which was confirmed by standard WHO insecticide susceptibility tube assays performed in June 2019 (<25% mortality 24 h after 1-h exposure to 0.75% permethrin or 0.25% deltamethrin-treated papers; data not shown here). The trials on these three islands were set up to determine if mass trapping (with varying trap densities) combined with LSM can eliminate mosquito populations and thus the nuisance and disease risk that they pose.

## 2. Materials and Methods

### 2.1. Study Island #1: Kunfunadhoo

**Figure 2:**
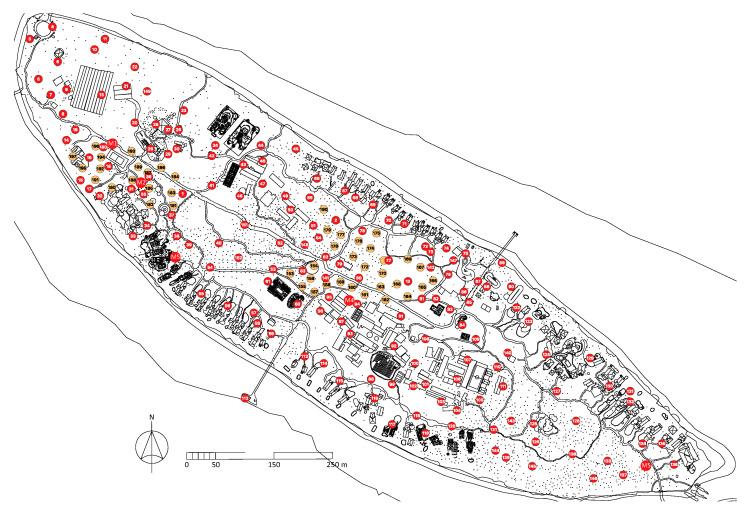
BG-MosquitaireCO2 trap locations on Kunfunadhoo island. Red numbered traps (150) were deployed in June 2019. Orange numbered traps (45) were deployed in October 2019 and M1-M5 (groups of 11 traps each; 55 in total) in March 2020, giving a total of 250 traps. For every numbered trap shown in red, 2 BG-GAT traps were deployed in their immediate vicinity (300 in total), in June 2019. Black dots on the map depict palm trees (*Cocos nucifera* L.).

In lieu of insecticide spraying, 150 BG-MosquitaireCO2 traps, which attract host-seeking mosquitoes with carbon dioxide and lactic acid, were deployed across Kunfunadhoo island in June 2019. In October 2019 and March 2020, an additional 45 and 55 traps were deployed, respectively (Figure 2), raising the average density of traps for host-seeking mosquitoes to 6.0 traps/ha. In addition, 300 BG-GAT traps, which lure gravid female mosquitoes seeking potential breeding sites to lay their eggs, were deployed in June 2019 (average density 7.2 traps/ha).

### 2.2. Study Island #2: Medhufaru

The second island, Medhufaru island (for a Google Earth image and trap locations, see Supplementary Material Figure S1), is located 70 km to the north-east of Kunfunadhoo, in the Noonu atoll. It is slightly larger (49.0 ha), has a maximum population of 700 staff and guests during full occupancy (Soneva, unpublished data), and the same two mosquito species are present. Interestingly, on Medhufaru island, *Cx. quinquefasciatus* bites humans, whereas this is not the case on Kunfunadhoo island. Starting in June 2020, a similar operation as described for Kunfunadhoo was launched on Medhufaru island (‘Soneva Jani’), with the exception that no cleanup operation was performed on the island prior to the start of or during the trial. Trap densities for the BG-MosquitaireCO2 traps and BG-GAT traps were 4.1/ha and 8.2/ha, respectively. As for Kunfunadhoo island, fogging against mosquitoes was terminated at the start of the trial. Since implementation of the same trapping/LSM strategy commenced a year later than on Kunfunadhoo, this enabled comparisons between both islands in terms of impact.

### 2.3. Study Island #3: Thahigandu Kolhu

The third island, Thahigandu Kolhu, is a small island 1.2 km north of Medhufaru island. It is 1.6 ha in size, is uninhabited and is used for day visits of guests for picnics (for a Google Earth image and drone video of the island, see Supplementary Material Figure S2 and Video S3, respectively). Similar to the first two islands, Thahigandu Kolhu boasted a large population of the same two mosquito species mentioned above and the elimination trial started in March 2021, when we deployed 10 BG-MosquitaireCO2 traps (density 6.3/ha), and we increased the density to 7.5 in September 2021 and subsequently to 18.8/ha in December 2021.

In addition to high-density trapping, larviciding on Thahigandu Kolhu was added from October 2021 onwards due to the very large number of crab holes that were frequently flooded and served as breeding sites for mosquitoes. We treated these and other water bodies once per week using Vectobac^®^ WG, a water-dispersible granule formulation of *Bacillus thuringiensis subsp. israelensis* (strain AM65-52), using an Oleo-Mac AM162 mistblower (Emak S.p.A, Bagnolo in Piano, Italy).

### 2.4. Mosquito Traps and Odor Baits

**Figure 3:**
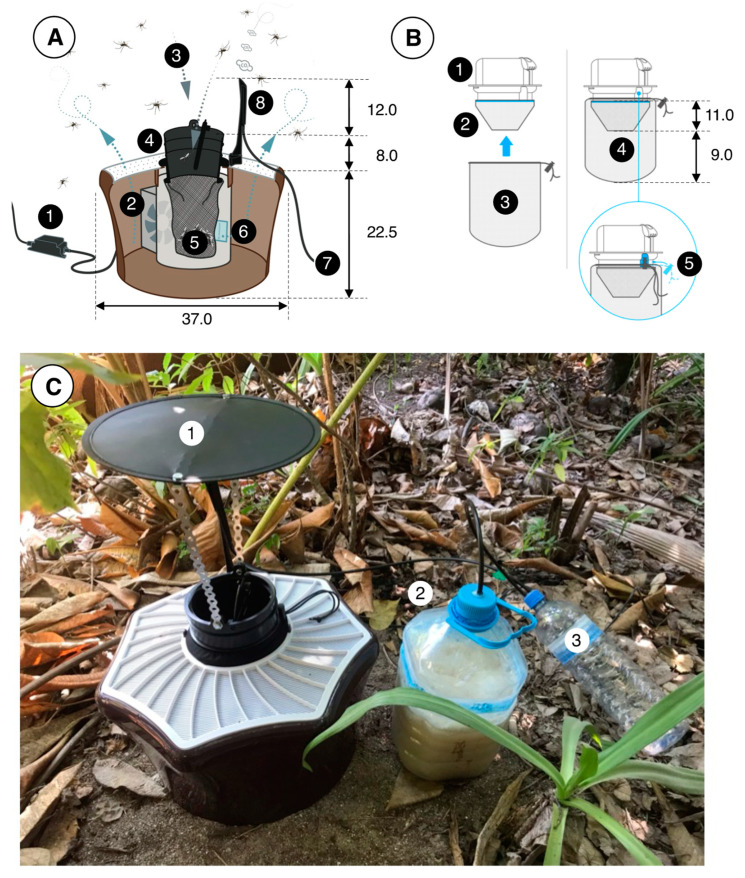
The BG-MosquitaireCO2 trap and its operation. (**A**): An adapter (1) converts power from the mains to 12 V DC (0.3 A, 3.6 W), which provides power to a fan (2) located inside the trap. Suction (ca. 3 m/sec) by the fan creates an inward airflow (3) through the black trap inlet (4) and the catch bag (5, see (**B**)) and odor-laden air (with lactic and hexanoic acid emanating from a sachet) (6) leaves the trap via its perforated white top. Carbon dioxide is provided though 6 mm plastic tubing (7) and released from a nozzle at the top of the trap (8). (**B**): The inlet (1) of the trap has two netting bags attached to it, a so-called funnel bag (2), which prevents mosquitoes from flying upwards, and the catch bag (3). Both bags (4) are fitted on the inlet (5) using an elastic string. (**C**): A trap in the field, fitted with a roof (1) to prevent rain from entering the trap. The 5 L water bottle (2) that contains 3 L of water, 700 g of sugar and 20–40 g of yeast to produce CO_2_ is located next to the trap. A 1.5 L overflow bottle (3) prevents liquid/foam from this mixture from entering the tubes and nozzle, which might otherwise become clogged and disrupt the flow of CO_2_. Tubes were fitted to the polyethylene bottle tops using hot glue. All dimensions are in cm. Image source: (**A**,**B**) Biogents AG, Germany; (**C**) B.G.J.K.

BG-MosquitaireCO2 traps (Figure 3) (Biogents AG, Regensburg, Germany) lure mosquitoes from within a range of ca. 20 m downwind of the trap. Mosquitoes fly upwind in the trap’s odor plume, and once they arrive in the vicinity of it, they become visually attracted by the contrasting colors of the trap components, notably the white cover top and central black inlet of the trap. When close to the trap, mosquitoes are sucked into the central black inlet through air suction produced by a 12 V DC fan located inside the trap and are then caught in a netting catch bag, where they die of dehydration [19] (Supplementary Material Video S4).

In order to attract host-seeking *Ae. albopictus* and *Cx. quinquefasciatus* females, as well as mate-seeking male *Ae. albopictus*, BG-MosquitaireCO2 traps were baited with sugar-fermenting yeast as an organic source of carbon dioxide (CO_2_) [20,21]. For every trap, a 5 L plastic water bottle was filled with 3 L of water, to which 700 g of white (granulated) sugar and 40 g of baker’s yeast (Bruggeman, Belgium) were added. This bottle was connected to a 1.5 L overflow bottle through polyethylene tubing (6 mm internal diameter) that in turn was connected to the CO_2_ outlet of the trap. The overflow bottle prevents foam, which often develops shortly after the fermentation process starts, from clotting the nozzle of the CO_2_ emitter. Carbon dioxide measurements revealed that this mixture produces a concentration of ca. 70 mL/min after 12 h (Supplementary Material Figure S3); thereafter, the concentration declines in a near-linear fashion. When a smaller inoculum of 20 g instead of 40 g of yeast was used, results were identical. Therefore, as of April 2020, all traps were baited with 20 g of yeast. This mixture would produce 11–13 mL/min of CO_2_ after 72 h, when it would be replaced. Even the peak concentration of ca. 70 mL/min after 12 h was not considered optimal in a recent study [22] that looked at the catch sizes of *Ae. albopictus* for different concentrations of CO_2_ when provided from a CO_2_ gas cylinder; as speculated before [20], catches may increase due to other (hitherto unidentified) attractants in the sugar/yeast fermentation mix.

A second attractant (BG-Mozzibait^®^, Biogents AG, Regensburg, Germany) consisted of a sachet filled with beads impregnated with a mixture of lactic acid [23,24], ammonium bicarbonate, hexanoic acid and other ingredients. These sachets were placed inside the trap and were replaced on a bimonthly basis from June to December 2019 and thereafter every month until June 2020, after which we increased the frequency of replacement to every fortnight.

Traps were deployed according to manufacturer recommendations. Traps were never placed in open sunlight but generally in shaded areas under vegetation cover. Every trap was visited every third day (Supplementary Material Figure S4); the catch bag was removed, labelled and replaced with a new catch bag. Catch bags were then placed in a −5 °C freezer for 45–60 min to kill any remaining live mosquitoes, after which catches for every trap were counted by species and recorded in an online database (Supplementary Material Data S1). During the entire operation, traps rarely suffered from malfunctioning, which was mostly due to power failure; on average, (±SD) 96.5 ± 2.9% of the traps were in good working condition when inspected (Supplementary Material Figure S5).

**Figure 4:**
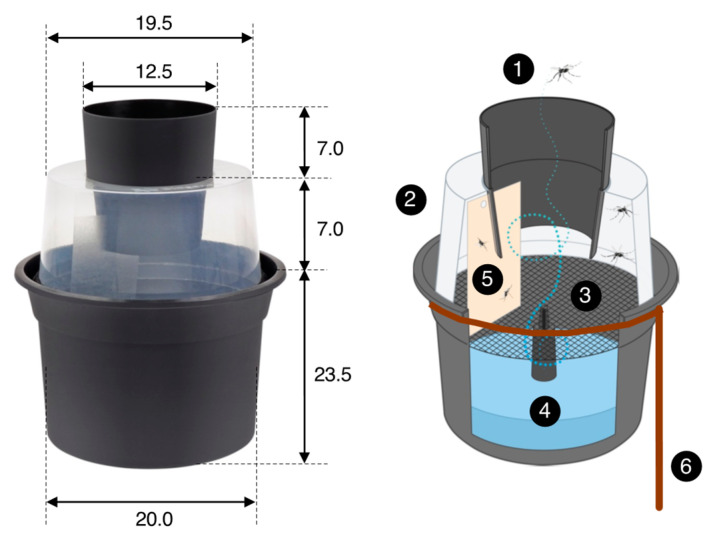
The Gravid Aedes Trap (or BG-GAT trap) is a passive trap that collects mosquitoes ready to lay their eggs. Female mosquitoes enter the trap through a black cone (1) and enter into a translucent chamber (2). A black nylon screen (3) separates the chamber from the oviposition substrate (4) (water with some organic material, e.g., some leaves). Unable to land on the water surface, mosquitoes fly around in the translucent chamber, where they collide with or land on a sticky card (5) (size 8 × 14 cm) on which they become trapped. A rebar wire (diameter 6 mm) ring with a peg (6) was used to position the trap solidly on the ground and prevent it from falling over. All dimensions are in cm. See Eiras et al. [23] for further details; image source: Biogents AG, Germany.

The Gravid Aedes Trap, or GAT (Figure 4), was developed to attract gravid female mosquitoes ready to lay eggs in standing water [25,26]. The GAT concept relies on visual and olfactory cues to lure gravid mosquitoes and a sticky card to trap and kill mosquitoes.

Gravid mosquitoes are lured to a black bucket base filled with rainwater, to which some leaves or organic debris is added to serve as an oviposition attractant. Once mosquitoes enter through the black inlet of the trap and enter the translucent chamber, they cannot directly come into contact with the water due to the presence of nylon netting material. At times, females succeed in passing eggs through this netting, in which case larvae can develop but adult mosquitoes emerging from pupae cannot leave the trap. The presence of (older) larvae has been shown to attract gravid females, as this may provide information about the suitability of the site to provide offspring [27,28]. Female mosquitoes flying around in the translucent chamber, when trying to escape from the trap, land on or collide with the sticky card and become trapped. Two BG-GAT traps were placed in the vicinity of every BG-MosquitaireCO2 trap deployed in June 2019. Sticky cards were replaced every fortnight, and the number and species of mosquitoes collected were recorded in an online database (Supplementary Material Data S2). If, at the time of servicing, a trap no longer contained water (e.g., during the dry season), it was replenished.

### 2.5. Egg Survival Experiments

In order to determine how long embryos of *Ae. albopictus* could survive inside eggs under dry conditions—a period that needs to be bridged even when elimination seems apparent [29,30]—we collected wild host-seeking females as they came to bite, using suction tubes to collect these upon landing on our legs and feet. Collected specimens were kept in 15 × 15 × 15 cm cages and were maintained on a 10% glucose solution. Every morning, the females were offered blood from the hand/forearm of B.G.J.K. for 10 min. Petri dishes with wet cotton wool and dark carton or pieces of wood were used to induce egg laying on these substrates. A total of 3840 eggs laid were removed individually with a fine brush and transferred to dry Whatman filter paper (20 eggs per piece of paper). These papers were kept inside Petri dishes and stored under ambient climatic conditions (temperature range 27–31 °C, RH 70–90%). Every week, for 26 weeks in total, 140 eggs (7 pieces of paper with 20 eggs each) were submerged in collected rainwater (inside a Petri dish) to which a small amount of yeast was added. After a week, the developing larvae were counted for every dish, totaled and converted to percentage hatch.

### 2.6. Heatmaps

Spatial interpolation with inverse distance weighting (IDW) was used to develop weekly or monthly heatmaps. IDW interpolation is a deterministic spatial interpolation approach that was used here to estimate mosquito abundance in locations without traps using actual catches from traps in the vicinity with corresponding weighted values. The basic IDW interpolation formula is:

(1) $$y=\frac{w_{1}x_{1}+w_{2}x_{2}+w_{3}x_{3}+\ldots +w_{n}x_{n}}{w_{1}+w_{2}+w_{3}+\ldots +w_{n}}$$

where *y* represents an unknown value at a given location, *w* is the weight, and *x* is the catch of a trap in the vicinity. The weight is the inverse distance of a point to each known trap catch that is used in the calculation. Simply, the weight can be calculated as follows:

(2) $$w_{i}=\frac{1}{d_{iy}^{P}}\;$$

where *d_iy_* is the distance to position *y* determined from, for instance, nearby trap locations 1, 2 and 3, with the distances to *y* point being *d*_1*y*_, *d*_2*y*_ and *d*_3*y*_. *P* represents a power value based on the lowest derived Root Mean Square Error (RMSE) between the interpolation result and the actual sampling value (here, we used *P* = 2). Using Equation (2), each respective weight could be calculated and then the value at position y was determined using Equation (1). QGIS software (QGIS.org, Grüt, Switzerland, version 3.8) was used to construct the maps based on the above IDW interpolation approach.

### 2.7. Larval Source Management

Larval source management (LSM) refers to the targeted management of mosquito breeding sites, with the objective to reduce the number of mosquito larvae and pupae. According to WHO [31], there are four main types of LSM: (1) habitat modification, which means a permanent alteration to the environment, e.g., land reclamation or surface water drainage; (2) habitat manipulation, which refers to a recurrent activity, e.g., water-level manipulation, flushing of streams, the shading or exposure of habitats; (3) larviciding, which involves the regular application of a biological or chemical insecticide to water bodies; and (4) biological control, which refers to the introduction of natural predators into water bodies—for example, predatory fish or invertebrates. On Kunfunadhoo island, we practiced types 2 and 3. Habitat manipulation was practiced by constructing roofs over areas where objects could hold rainwater and provide breeding sites. All large trees on the island were examined for the presence of ponds (natural water bodies where tree branches meet); these were inspected and filled with sand on a monthly basis. Similarly, all septic tanks on the island were geo-referenced (Supplementary Material Figure S6) and inspected monthly. Lids of septic tanks were sealed using cement after every inspection.

Besides trapping, the island was subjected to a massive cleanup operation of potential breeding sites in June 2019 and again in July 2020. For a period of 3–4 weeks, a team of 15 casual laborers would move through the jungle to remove any potential breeding sites. More than 190 thousand potential breeding sites (empty coconuts, coconut spathe bowls (‘coconut boats’), plastics and glassware) were removed from the jungle and shredded in 2019, and half this number in 2020 (Supplementary Material Figure S7). From August 2019 onwards, team members would inspect one or more of 40 blocks (Supplementary Material Figure S8) into which the island was divided on a weekly basis. They would spend 2–2.5 h per block, systematically scanning the terrain for any possible water body that could serve as a mosquito breeding site. Transient water bodies, such as empty coconuts or coconut boats holding rainwater, would be removed or turned upside down. Non-natural materials holding water (e.g., plastics, tarpaulins, bottles, etc.) would be removed altogether (Supplementary Material Figure S9).

For larviciding, we used a botanical insecticide based on neem oil (*Azadirachta indica*; NatureMate (Pvt) Ltd., Dankotuwa, Sri Lanka) [32]. This oil is an emulsified concentrate that contains 35% neem seed kernel (equivalent to 0.15% azadirachtin) extracted in 60% of solvent methanol and 5% emulsifier (polysorbate 20). Oil was mixed with water to obtain solutions of 4, 100, 400 or 800 mL/L, which were applied on water surfaces using a knapsack sprayer (1–3 swaths for all treatments; controls were sprayed with water only). Larvae used in the experiments (50 3rd/4th instar larvae per replicate; each concentration was replicated 3 times) were collected from coconut boats (see Supplementary Material Figure S9) in the jungle in which larvae were observed. These were transferred to containers, morphologically separated by species and then returned to the original coconut boat. No additional food was provided during the experiment. Subsequently, larvae were observed for up to 120 h post exposure to the neem oil treatments; pupation and emergence of adult mosquitoes was recorded.

For experiments with seawater, 3rd/4th instar larvae were collected from a variety of breeding sites, morphologically identified to species and transferred to 500 mL plastic containers that contained the following seawater/freshwater proportions (%): 100–0, 75–25, 50–50, 25–75, 0–100 (control). Twenty-five larvae per concentration, replicated 6 times, were tested, and larval mortality, pupation and emergence recorded for up to 120 h post exposure of larvae to the treatments. Larvae were provided with some dead leaf material and biscuit powder as a source of food. Pairwise comparisons between the means for treatments for both the neem oil and seawater experiments were performed using Wilcoxon signed rank tests (considered significant at *p* < 0.05).

### 2.8. Operations and Direct Cost Analysis

The field operation was managed by A.J., integrated pest control manager at Soneva (0.8 FTE dedicated to the mosquito elimination project). He coordinated all field activities undertaken by a team of 6 assistants (6 FTE). On a daily basis, 2 of these assistants would visit ca. 80 Day 1, Day 2 or Day 3 traps (Supplementary Material Figure S4) to replace bottles with water/sugar/yeast for CO_2_ production; the other 4 staff would be involved in LSM activities or general duties related to the project. The rationale for servicing traps in this manner was based on the production of CO_2_, which declines after one day (Supplementary Material Figure S3). Rather than servicing 80 traps in one part of the island, with the risk of having low concentrations of CO_2_ for all traps in that area two days afterwards, it was decided to intersperse traps receiving fresh bottles with the water/sugar/yeast mix with traps that received these one or two days prior. In this way, although, in terms of time spent servicing and distance covered, this was less economical, the chance of mosquitoes encountering a trap with a high(er) concentration of CO_2_ was increased. Field staff would use a battery-operated buggy to move around on the island. Besides refreshing the CO_2_ source, they would collect catch bags from every trap and transport these to the laboratory for counting. All assistants were trained in mosquito identification. Pre-printed forms were used for recording trap catches and data were subsequently transferred to an online Excel database (Supplementary Material Data S1). Every fortnight, the team would also inspect the sticky cards inside the BG-GAT traps for the presence of mosquitoes and record numbers on pre-printed datasheets and subsequently in an online Excel database (Supplementary Material Data S2).

Prior to the current project, a professional pest control company was responsible for controlling mosquitoes on the island. Under a contract that amounted to USD 110,000 per year, the company only practiced misting or hot fogging with synthetic pyrethroid insecticides and did not undertake any LSM. This contract ended in March 2019, when outdoor insecticide spraying on the island was terminated. From June 2019 onwards, when trapping and LSM replaced insecticide spraying, direct cost estimates of the replacement strategy were kept in order to contrast these with the third-party contract.

## 3. Results

**Figure 5:**
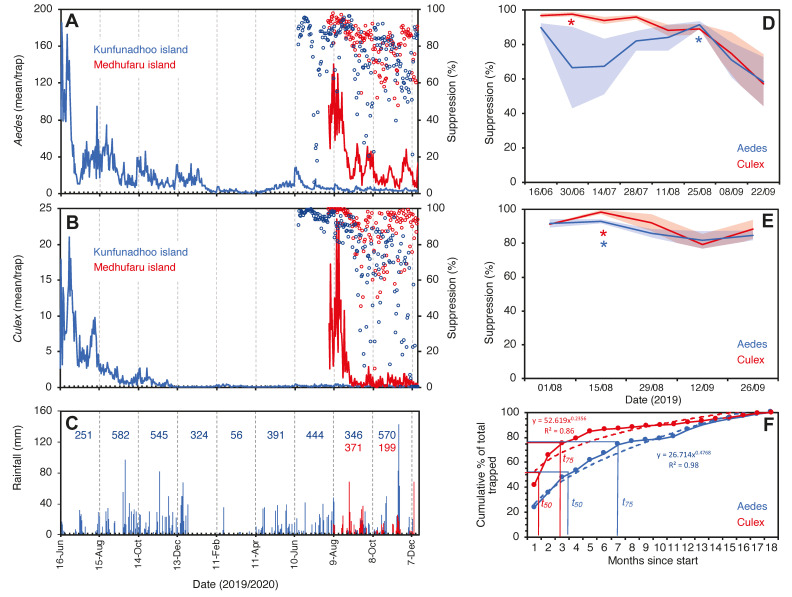
Trap catches of *Ae. albopictus* (**A**) and *Cx. quinquefasciatus* (**B**) mosquitoes in Kunfunadhoo (blue line) and Medhufaru (red line) islands, Maldives, between June 2019 and December 2020. Open blue circles in both graphs show the percentage suppression of mosquito populations compared to the same date one year previously. Open red circles show the percentage suppression on Kunfunadhoo island compared to Medhufaru island. (**C**): Actual rainfall (bars) and cumulative rainfall over two-month periods (numbers) for Kunfunadhoo (blue) and Medhufaru island (red). (**D**): Level of suppression (percentage ± 95% CI) compared to one year previously (asterisks (*) show highest level recorded for *Aedes* (red) and *Culex* (blue) mosquitoes). (**E**): Similar, but between-island comparison of suppression levels. (**F**): Cumulative percentage of mosquitoes removed from Kunfunadhoo island over the entire 18-month period, showing the time needed to trap 50% (*t*_50_) or 75% (*t*_75_) of all mosquitoes collected for both species.

On Kunfunadhoo island, within a month after the deployment of the traps and the jungle cleanup activities, catches for both mosquito species (Supplementary Material Data S1, S2) dropped sharply to much lower numbers (Figure 5A,B; for GAT trap data, see Supplementary Material Figure S10). In the first month alone, traps for host-seeking mosquitoes caught a total of 113,085 *Ae. albopictus* and 14,559 *Cx. quinquefasciatus* (Supplementary Material Video S4), and of all the mosquitoes collected over the entire 18-month trial period (475,224 *Ae. albopictus* and 34,660 *Cx. quinquefasciatus* specimens), 75% had been trapped by month 7 for *Aedes* and month 3 for *Culex* (Figure 5F), which indicates a rapid and significant impact of trapping and LSM on the mosquito population (power function for removal of the *Aedes* population per month *t* as per the following formula).

% Removal _(*t*)_ = 26.7 (*t*)^0.48^ (R^2^ = 0.98);

The same power function for *Culex* is

% Removal _(*t*)_ = 52.6 (*t*)^0.24^ (R^2^ = 0.86).

**Figure 6:**
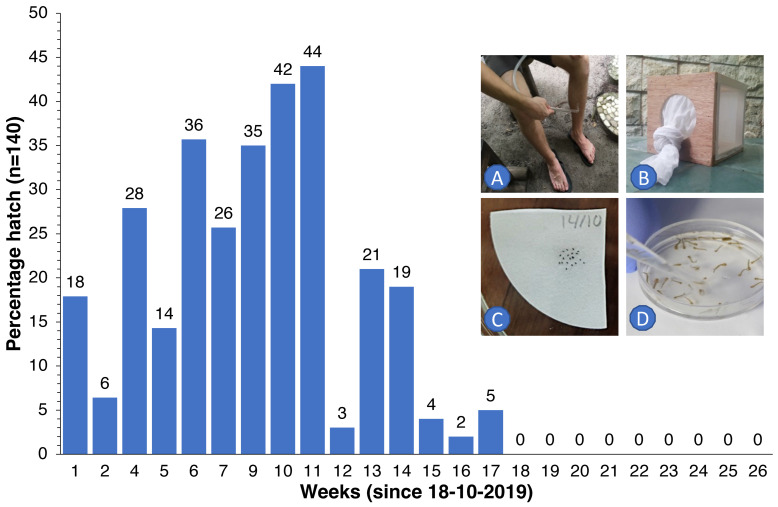
Percentage of *Ae. albopictus* eggs (for every week, *n* = 140) that hatched following submergence in rainwater after 1 to 26 weeks of storage under dry ambient conditions. Wild females were collected with a suction tube as they came to bite (**A**) and were then kept in 15 × 15 × 15 cm cages (**B**), blood-fed and offered a substrate to lay their eggs. Eggs were then individually placed on Whatman filter paper ((**C**), 20 eggs per paper) for storage. At weekly intervals, 7 papers with eggs were submerged in water to determine how many eggs would hatch and develop into larvae (**D**).

We observed that eggs of local *Ae. albopictus* could survive in the absence of water for up to 5 months (Figure 6). Therefore, continuous replenishment of the population following rainfall and gradual depletion of the ‘egg bank’ took almost 6 months. Thereafter, coinciding with the onset of the dry season (Figure 5C), the population was further reduced to near elimination levels and did not recover to pre-intervention levels in spite of the arrival of heavy monsoon rains in April 2020. For *Culex*, the drop was more pronounced and faster since egg rafts of this species are not drought-resistant. A small peak in *Aedes* numbers was observed in June 2020 (Figure 5A), when the upkeep of villa pools and ponds was temporarily suspended due to the absence of guests in the resort because of the COVID-19 pandemic, which turned many of these into potential breeding sites after filling up with rain. *Culex* numbers, nevertheless, remained low throughout this period (Figure 1C).

**Figure 7:**
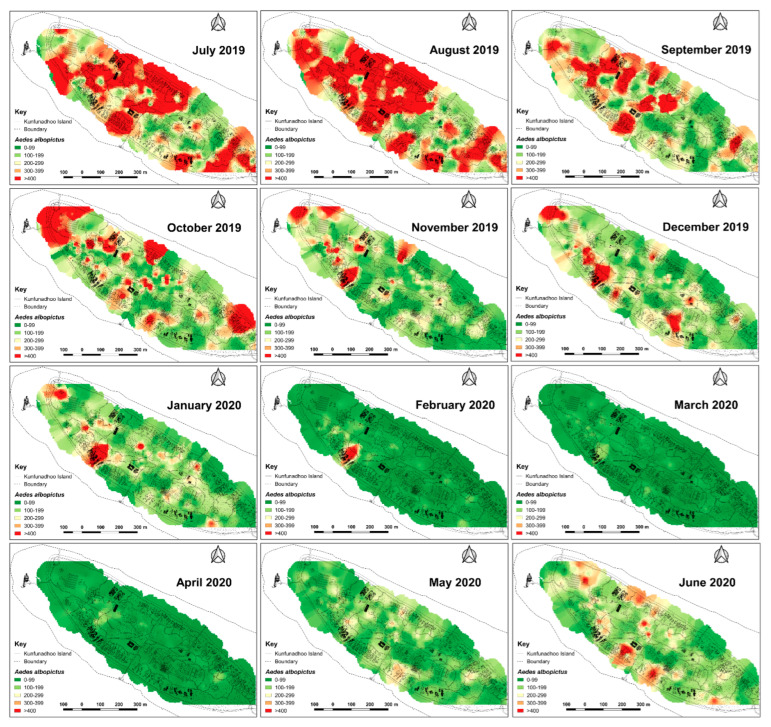
Inverse distance weighted heatmaps of *Aedes albopictus* showing areas with high (red) or low (green) mosquito catches by month for the period June 2019–June 2020. Following the first three months, with high densities across the northern part of the island, more discrete foci with high numbers emerged from October 2019 onwards, eventually resulting in very low numbers during the peak of the dry season (April 2020). Thereafter, notably in June, numbers increased when maintenance of potential breeding sites such as pools and ponds was temporarily suspended (due to COVID-19).

**Figure 8:**
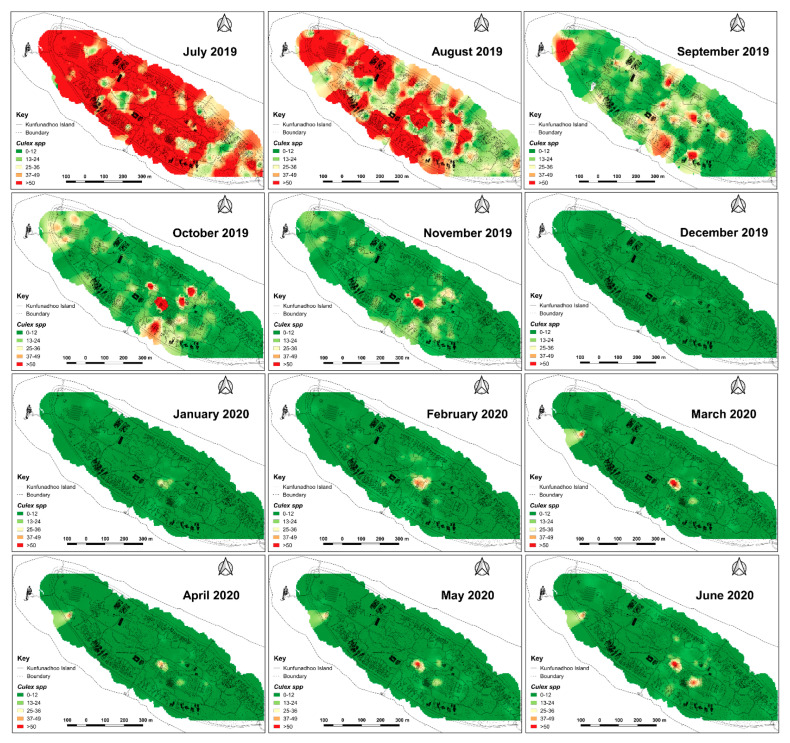
Inverse distance weighted heatmaps of *Culex quinquefasciatus* showing areas with high (red) or low (green) mosquito catches by month for the period June 2019–June 2020. Impact of trapping was observed faster than for *Ae. albopictus* (Figure 7) since *Culex* eggs cannot survive a period of drought.

Since traps on Kunfunadhoo island were numbered and deployed across the island, weekly or monthly heatmaps were constructed for both species (Figure 7 and Figure 8), which were used to guide additional trap deployment as well as LSM. The impact of the intervention was contrasted with a ‘control’ in two ways. First, daily trap catches from 16 June 2020 onwards were compared with daily catches one year previously (the trial started on 16 June 2019), giving a ‘within-island’ comparison and therefore a daily percent suppression value compared to 1 year earlier. The highest level of suppression of the *Aedes* population compared to 1 year previously was recorded in August 2020 (91.4% (95% CI 89.1–93.8)) and for *Culex* in June 2020 (97.8% (95% CI 95.8–98.2)) (Figure 5D). Second, after a similar trapping operation was implemented on Medhufaru island (Noonu atoll), 75 km from Kunfunadhoo island, in June/July 2020, we could make ‘between-island’ comparisons of catches. In August 2020, the between-island comparisons showed that population suppression on Kunfunadhoo island was extremely high compared to Medhufaru island (for *Aedes*, 93.0% (95% CI 91.7–94.4); for *Culex*, 98.3% (95% CI 97.0–99.5)) (Figure 5E). Thus, both within- and between-island comparisons showed dramatic reductions in mosquito numbers, and this effect can be attributed to the interventions, considering that a key determinant of population size, namely rainfall (Figure 1C), was not notably different between years and islands. Thereafter, as expected, both within- and between-island comparisons showed lower suppression levels since mosquito populations declined due to the intervention.

**Figure 9:**
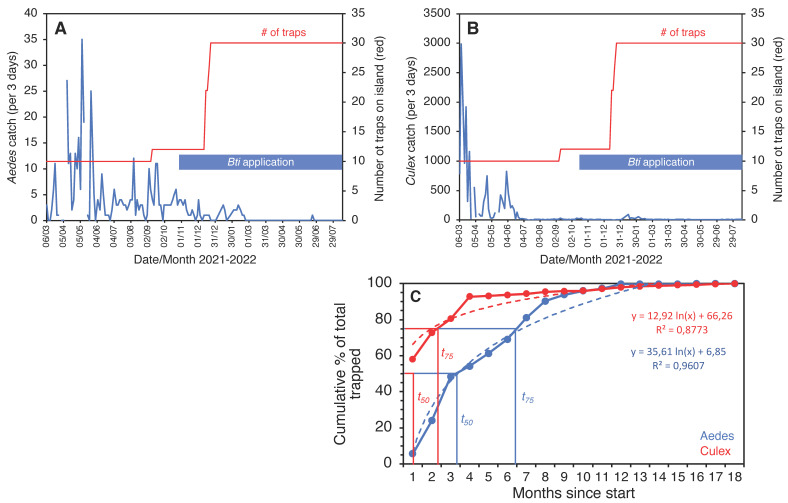
Three-day catches of *Ae. albopictus* (**A**) and *Cx. quinquefasciatus* (**B**) from Thahigandu Kolhu island between March 2021 and August 2022. Over time, the trap number (red line) was increased from 10 to 30, and larviciding with *Bti* (blue bar) was conducted weekly from 28 October 2021 onwards. Elimination of *Aedes* mosquitoes was accomplished on 20 February 2022. One mosquito was caught at the end of June but identification was inconclusive. Gaps in the blue line graphs indicate absence of data (due to lack of electricity on the island). Graph (**C**) shows the cumulative percentage of mosquitoes removed from the island over the entire 18-month period, showing the time needed to trap 50% (t_50_) or 75% (t_75_) of all mosquitoes collected for both species.

The trial on the smallest island, Thahigandu Kolhu, which started in March 2021, showed a similar sharp decline in mosquito numbers (Figure 9). The initial trap density of 6.3 traps/ha was clearly sufficient to drastically lower the *Culex* population, but, for *Aedes*, this was less pronounced and only started when the density exceeded 7.5 traps/ha. When the density reached 18.8 traps/ha, it took 8 more weeks until the *Aedes* population was eliminated. Ironically, even though *Culex* numbers dwindled rapidly and by >99.7% compared to catches at the start of the trapping operation, the species persists in single digit numbers and is not yet eliminated. It is encouraging, nevertheless, to see that the speed at which populations were removed from the island (Figure 9C) is strikingly similar to that observed for Kunufadhoo island (Figure 5F). On both islands, it took less than 3 months to remove 75% of all *Cx. quinquefasciatus* mosquitoes trapped over the entire 18-month trapping period, and this was less than 7 months for *Ae. albopictus*.

**Figure 10:**
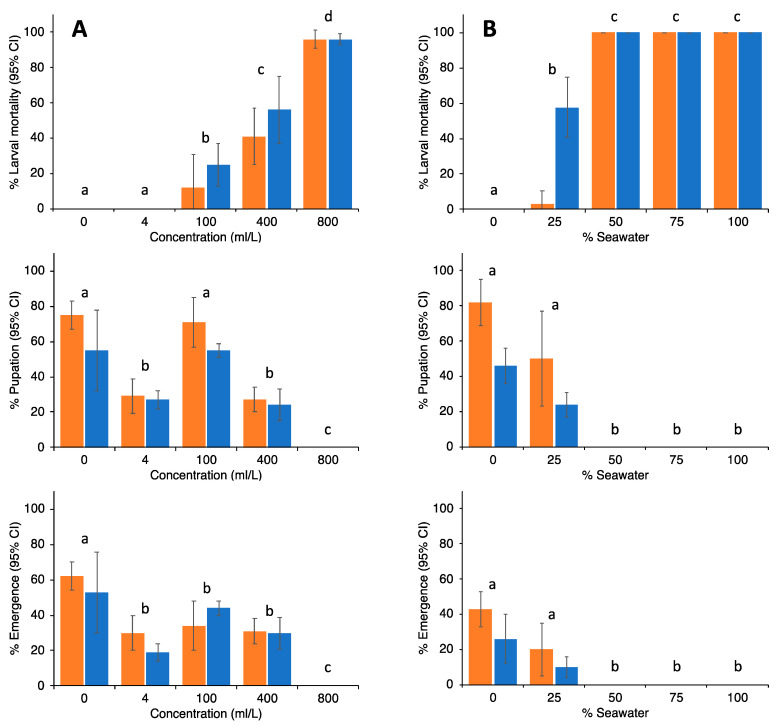
Percentage (±95% CI) of larval mortality, pupation and emergence of *Ae. albopictus* (orange bars) and *Cx. quinquefasciatus* (blue bars) when exposed to different concentrations of neem oil ((**A**), left graphs) or seawater ((**B**), right graphs). No significant differences were observed between both mosquito species. Differences between treatments are indicated by letters; treatments with different letters are significantly different at *p* < 0.05 (Wilcoxon signed rank tests).

Results from the trials with neem oil and sea water as potential larvicides are shown in Figure 10. For neem oil, complete prevention of adult emergence was observed at 800 mL/L. For seawater, any solution containing more than 50% seawater would completely prevent emergence. In particular, seawater, since it is readily available, has been used in ponds and swimming pools (for instance, see Figure S7, panel 3), and they have been mosquito-free ever since.

A direct cost analysis (Supplementary Material Table S1) showed that the first year of operation cost USD 85,123, or USD 2056 per hectare. The bulk of these costs were labor (51.5%), followed by BG-MosquitaireCO2 trap attractants (40.8%; notably the cost of BG-Mozzibait^®^ and sugar). The contract previously held with a commercial pest control firm amounted to USD 110,000 per annum, so trapping/LSM was 22.6% cheaper than the insecticide-based option (which was ineffective due to insecticide resistance).

## 4. Discussion

Our findings indicate that trapping, combined with LSM, can reach population suppression levels comparable with the novel genetic control strategies piloted over the last decade. On Thahigandu Kolhu island, we actually exceeded this by demonstrating 6+ months of elimination of *Ae. albopictus*. We acknowledge two shortcomings of our trials. First, it is impossible to have ‘control’ treatments. Not every island is the same, and direct comparisons of effects must be interpreted with care. Second, due to the inherent nature of the trial, we acknowledge that deciphering the actual contribution of the specific components of the intervention (traps for host-seeking mosquitoes, traps for gravid mosquitoes and LSM) is impossible. The only argument in support of trapping is that Kunfunadhoo attempted to reduce its mosquito populations through intense LSM (in 2014 and again in 2016) but did not reach anywhere close to current levels of suppression. This supports the view that trapping was the major contributor to the successes reported here. Finally, it is striking how similar the collapse of mosquito populations on all three islands was (Figure 5F and Figure 9C), and the speed at which these were removed was remarkably similar, adding confidence that this was not a random effect but actually caused by trapping/LSM.

We observed that, under prevailing conditions in the Maldives, eggs of *Ae. albopictus* could withstand desiccation for a period of 5 months. Although rainfall is present in every month of the year, a ‘dry’ season is experienced between December and April. In the absence of suitable breeding sites and standing water, eggs have to survive this period using dormancy [29]. Although we have no other data from the Maldives, Sota [30] reported survival periods of 95 days (at a relative humidity (RH) of 88%), 69 days at 68% RH and 35 days at 42% RH, for *Ae. albopictus* from Malaysia. A laboratory study with *Ae. albopictus* from Italy reported that a hatch rate above 80% was obtained after 11 weeks of conventional storage (on filter paper in a sealed bag) [33]. After this period, hatching decreased dramatically; no eggs hatched after 24 weeks. Storing eggs in water produced an 85% hatch rate after 5 months in both species. Our own data suggest that, in order to ensure that no eggs remain viable in the jungle to kick-start a new population, traps should remain operative for at least 6 months after catches drop to zero (as we experienced on Thahigandu Kolhu island).

In line with previous research on neem oil as a potent larvicide against mosquitoes, our findings here showed similar activity against both *Ae. albopictus* and *Cx. quinquefasciatus.* Neem oil is a relatively cheap insecticide (ca. USD 9/L) compared to the much higher costs of synthetic pyrethroid insecticides and could even be locally produced from neem seeds. The cheapest option that we now have is seawater. Apparently, tolerance for salinity in both species is surpassed in concentrations over 50% sea water. Especially during the dry season, when pond waters are not diluted by rainfall, this option is a highly practical means to prevent the breeding of mosquitoes.

Compared to contemporary genetic control strategies, our approach has several explicit advantages. Firstly, (a) trapping targeted both species of mosquito (*Aedes* and *Culex*) whereas genetic control trials target only one species; in addition, (b) trapping and LSM can be undertaken by personnel after only minimal training. Trap maintenance and developing a keen eye for finding potential breeding sites are skills that can be learnt with minimal effort. The approach can therefore easily be expanded to other parts of the world where these mosquito species prevail. This is very different for genetic control trials that require highly skilled personnel. Moreover, (c) trapping does not require the construction of mass-rearing facilities needed for genetic control trials, which are very expensive and again require specialized skills and expertise; (d) trapping is very much a ‘seeing is believing’ approach, and quickly results in positive responses from the community when seeing catch bags filled with large numbers of trapped mosquitoes. Genetic control trials, in contrast, are based on the release of mosquitoes in the environment, which certainly, in the case of genetically engineered mosquitoes, has resulted in incidents of strong public opposition to such trials; (e) trapping and LSM is significantly cheaper than genetic control trials. For instance, a recent trial in China, in which classical SIT was combined with the use of *Wolbachia*, over an area of 32.5 ha, over a 4-year period in which 197 million mosquitoes were released, suppressed the *Aedes* population by 96% [5]. The actual cost of this trial ranged from USD 54 to 216/ha/week, depending on the number of males released per hectare. This would translate into USD 116–465 thousand per year for a similar operation on Kunfunadhoo island. Trapping/LSM would thus be 26.6–81.7% cheaper than the genetic control option; (f) unlike genetic control trials, for which it often takes years to obtain regulatory approval, conduct community sensitization, produce mosquitoes and plan releases, mass trapping campaigns can be rolled out within a few months; and, finally, (g) given the rapid decline of mosquito populations after trap deployment, the approach can easily and quickly be adopted during arboviral disease outbreaks, which is not possible for genetic control options or insecticide spraying in areas where mosquitoes have developed resistance to approved insecticides.

It is highly encouraging that we succeeded with the elimination of *Ae. albopictus* from a small island. Even at a high trapping density and high initial cost, this can permanently end the risk of all mosquito-borne disease transmission and result in vastly positive payback over time. As of now, a trap density of 10–15 traps/ha is probably sufficient to reach elimination within 18 months of operation, although we acknowledge that this should be repeated on more islands. This is currently being investigated on Mudhdhoo island (Baa atoll) and Muravadhoo island (Raa atoll), each having 10 traps/ha. On a third island, Dipparufushi (which is only 3 ha in size), we are operating a density of 15 traps/ha in an ongoing attempt to eliminate both species.

Although the BG-MosquitaireCO2 traps have been shown to be highly effective in attracting mosquitoes over a distance, their actual trapping efficiency remains rather low (<5% of trap approaches resulting in capture), leaving significant room for trap design improvement [34]. Clearly, when trapping efficiency increases, fewer traps will need to be deployed per ha, reducing the cost even further.

Although trapping at high density in combination with LSM can be sufficient for elimination, integrated approaches may still be explored, whereby trapping/LSM is initially used to drastically reduce populations, followed by mass releases of *Wolbachia*-infected males (using cytoplasmic incompatibility as a means to drive sterility into the population) or sterile males to eliminate remaining foci. In other words, trapping/LSM can work in harmony with genetic control trials, and combined approaches may result in (faster) elimination, which, to date, none of these genetic control methods have succeeded in achieving.

A recurring issue is whether or not mosquitoes can easily re-invade islands once they have been freed of them—for instance, through boat or aircraft travel. Interestingly, the island neighboring Kunfunadhoo island at a 3.6 km distance, Maalhos, harbors a species of mosquito (*Armigeres obturbans*, Theobald) not found on Kunfunadhoo, in spite of daily boat travel to and from this island for many years. It is very likely that specimens of this species have successfully entered Kunfunadhoo island but that their minimum founder population size was always too small to sustain sufficient genetic heterogeneity to become successfully established. Perhaps only when a large number of eggs is introduced (e.g., through the introduction of used car tires in which many females have laid hundreds or thousands of eggs) could this be overcome and an invasion be successful.

Given the small size of the islands and their geographic isolation, which make these ideal for mosquito elimination, it remains to be seen if the same approach can result in similar impacts on the mainland. Although evidence is sparse at the moment, a study conducted in Southern France indicated that a high-density trap barrier placed around inhabited premises can result in the near elimination of *Ae. albopictus* inside this perimeter after a period of a few weeks [35].

Finally, a critique frequently heard is the potential consequences of removing mosquito species from ecosystems. Although there appears to be a consensus that their role in nature is fairly limited [36,37], this certainly appears to be the case on small Maldivian islands that, due to their porous coral soils, are largely void of surface freshwater and hence fish and amphibians that could predate on mosquito larvae. Obviously, the impact of chemical spraying, which is still the predominant practice of controlling mosquitoes in the Maldives, will be much greater and affect insect biodiversity to a much larger extent than the trapping of one or a few mosquito species. Within 1.5 years without spraying of insecticides in its jungle, Kunfunadhoo island experienced the return of a broad array of butterflies, dragonflies and bumble- and carpenter bees—all species that had all but disappeared from the island but are now restoring balance in the fragile island ecosystems witnessed on small islands. Moreover, ending the spraying of synthetic pyrethroids, the dominant insecticides used for fogging against mosquitoes in the Maldives, will benefit corals and marine life, for which these chemicals are extremely toxic. Trapping and LSM will therefore not only benefit terrestrial biodiversity and, in particular, insect diversity, which is globally under threat [38], but also result in the additional protection of vulnerable and endangered coral/reef ecosystems already under pressure due to climate change [39].

## 5. Conclusions

We have shown, for the first time, that human odor-baited mosquito traps, when used at high density and in combination with larval source management (LSM), can be used to sustainably eliminate or drastically reduce (93–98%) mosquito populations from small Maldivian islands within a period of 1.5 years. High-density trapping and LSM pose the first alternative in decades to manage mosquito-borne disease risk on small (tropical) islands in an affordable and environmentally friendly manner.

## Data Availability

Raw trapping data are provided in the Supplementary Materials, Data S1 (BG-MosquitaireCO2 trap data) and Data S2 (BG-GAT trap data).

## Supplementary Materials

The following supporting information can be downloaded at: https://www.mdpi.com/article/10.3390/insects13090805/s1, Figure S1: Location of Medhufaru island; Figure S2: Location of Thahigandu Kolhu island. Figure S3: Production of carbon dioxide (CO_2_) through sugar fermentation; Figure S4: Continuous 3-day routine inspection of traps, servicing and mosquito catch collection; Figure S5: The percentage of Day 1, Day 2 and Day 3 traps in good working order at the time of servicing; Figure S6: Fixed breeding sites targeted through larval source management (LSM). A: Locations of 56 tree ponds; B: Locations of 54 septic tanks; Figure S7: Removal of potential breeding sites from Kunfunadhoo island in 2019 (A) and 2020 (B); Figure S8: Division of Kunfunadhoo island into 40 sectors that are visited weekly to conduct larval source management (LSM); Figure S9: Examples of typical breeding sites that were removed during larval source management inspections; Figure S10: Absolute number of *Aedes albopictus* (in blue) and *Culex quinquefasciatus* (in red) collected from sticky cards of GAT traps over 2-week periods, July 2019–December 2020; Table S1: Direct costs for the first year of operation; Video S1: Impression of the dense tropical vegetation of Kunfunadhoo island, Baa atoll, Maldives. Video S2: The predominant method of mosquito control in the Maldives is hot fogging, whereby fast-acting pesticides such as pyrethroids are used. Video S3: 2972 mosquitoes (both *Aedes albopictus* and *Culex quinquefasciatus*) collected from a single BG-MosquitaireCO2 trap over a 24-h period. Video S4: Drone impression of Thahigandu Kolhu island (north of Medhufaru island). Data S1: BG-MosquitaireCO2 trap catch data for *Aedes albopictus* and *Culex quinquefasciatus* from June 2019 through December 2020. Day 1–3 trap numbers correspond to trap locations shown in Supplementary Material Figure S3. ND = trap not (yet) deployed; NF = trap not functional. Data S2: GAT trap catch data for *Aedes albopictus* and *Culex quinquefasciatus* from July 2019 through December 2020. Day 1–3 trap numbers correspond to trap locations shown in Supplementary Material Figure S4, but have ‘A’ and ‘B’ added to the number assigned to the BG-MosquitaireCO2 trap.
