# Supplementary material for: Mass Trapping and Larval Source Management for Mosquito Elimination on Small Maldivian Islands
Source: Insects. 2022 Sep 2;13(9):805. doi: 10.3390/insects13090805 (PMC9503984; doi:10.3390/insects13090805)
Supplement: Supplementary file 1 [file insects-13-00805-s001.zip › insects-1898599-supplementary/Figures S1-S10.pdf]

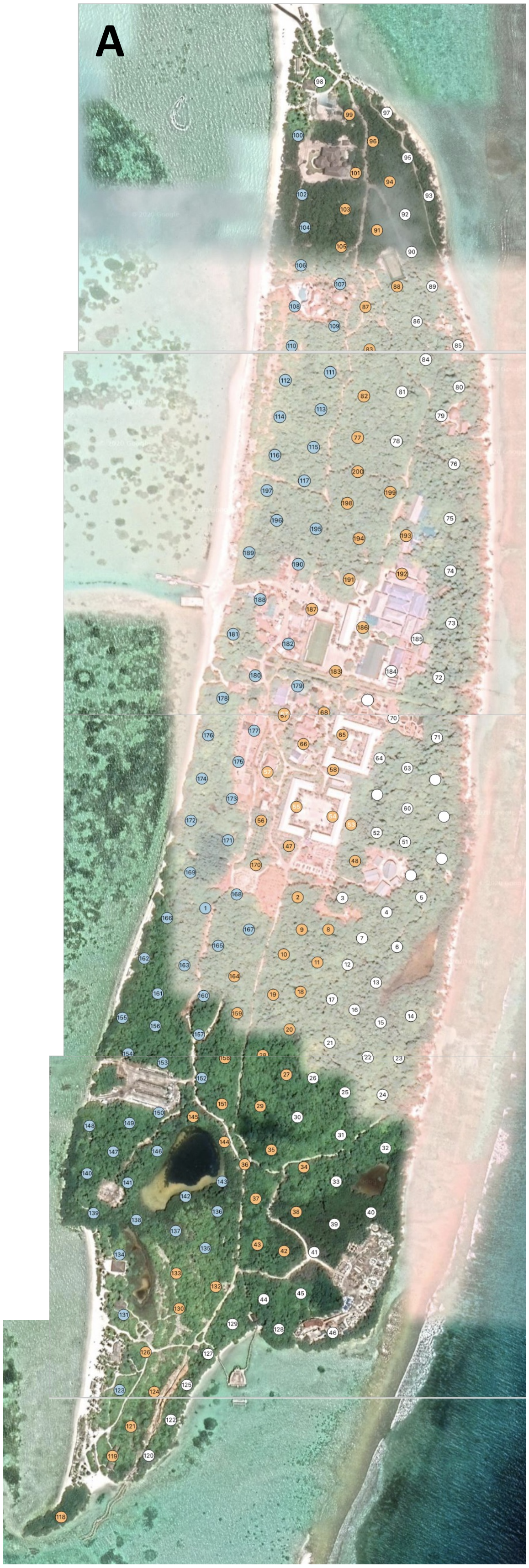

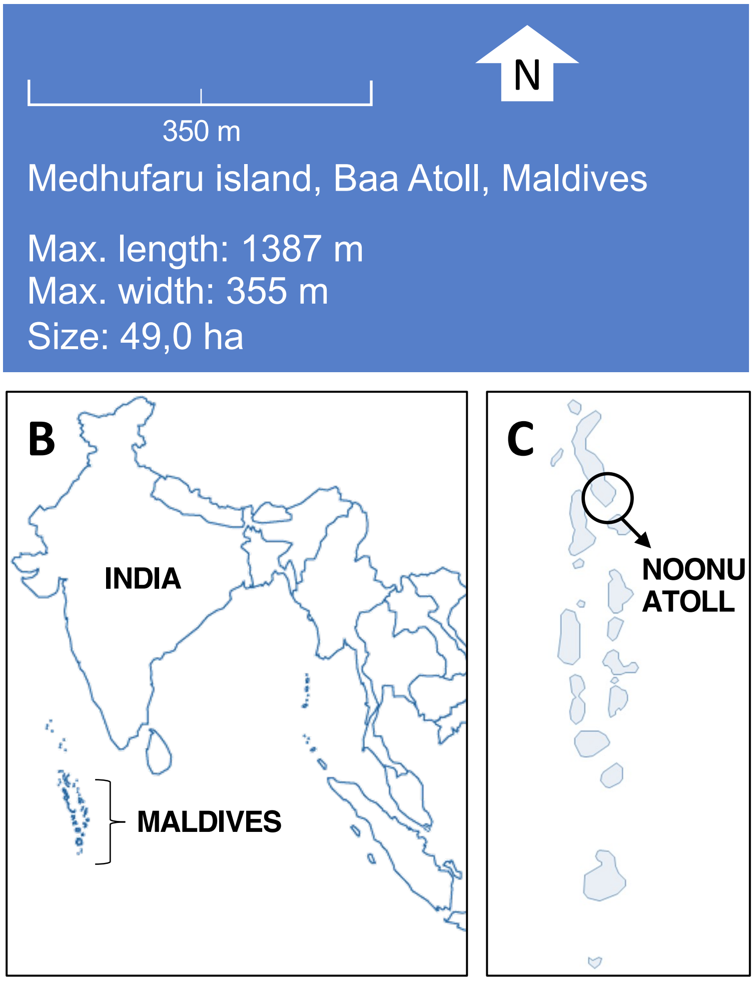

Figure S1. Location of Medhufaru island. A: Google Earth satellite image of Kunfunadhoo island (Imagery date 9/8/2019), showing the 200 BG-MosquitaireCO2 trap locations. Traps with the same colour are serviced on the same day. B: Location of the Maldives. C: Location of the Noonu Atoll. The geographical center of the island is located at 5° 42′ 48.73″ N, 73° 24′ 54.11″ E.

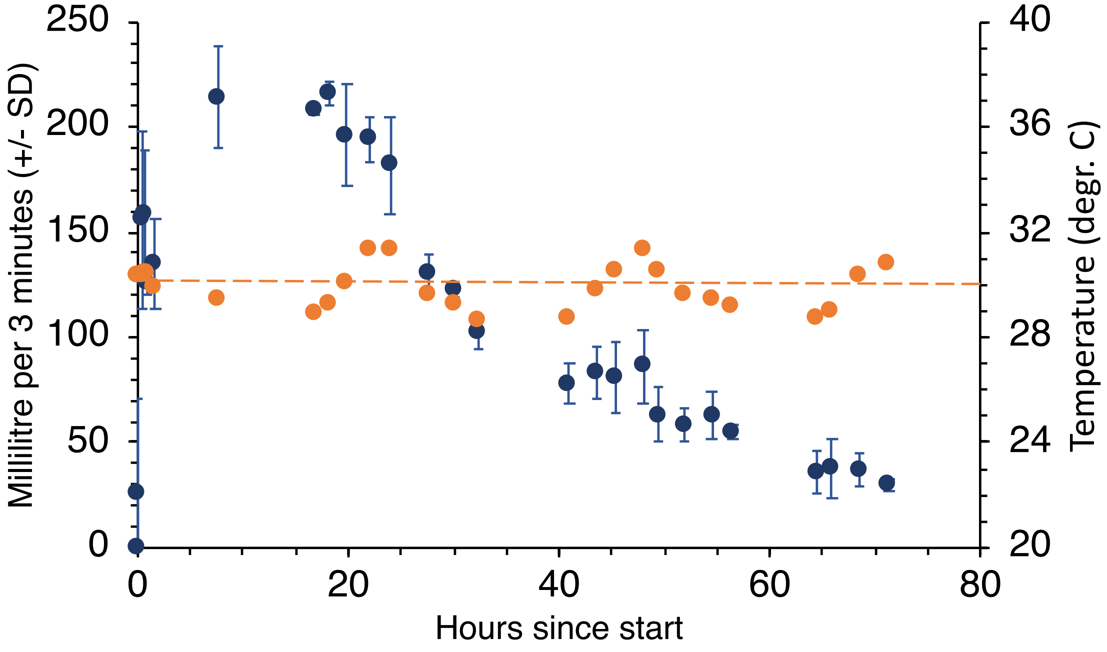

Figure S2. Production of carbon dioxide (CO_2) through sugar fermentation. A 5 L water bottle was filled with 3 L water to which 700 g of white sugar and 40 g of yeast was added. Three measurements were taken every time CO_2 production was measured (blue symbols; in ml per 3-min period ± standard deviation of the three measurements). Orange symbols indicate outdoor temperature at the time of measurement, which averaged at 29,9 ºC (dotted orange line).

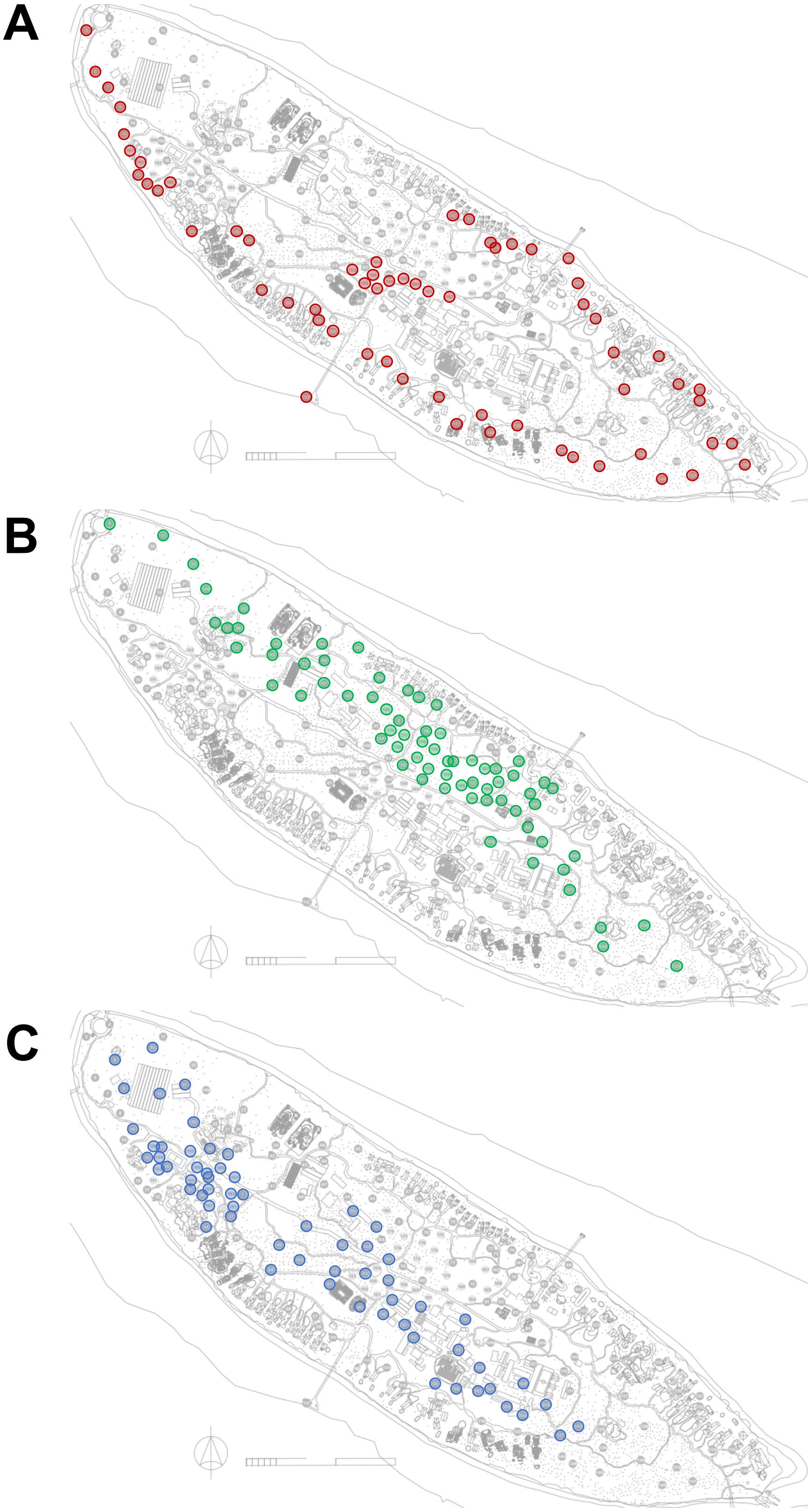

Figure S3. Continuous 3-day routine inspection of traps, servicing and mosquito catch collection. A: Traps inspected, serviced and emptied on Day 1, B: similar, on Day 2, and C: similar, on Day 3.

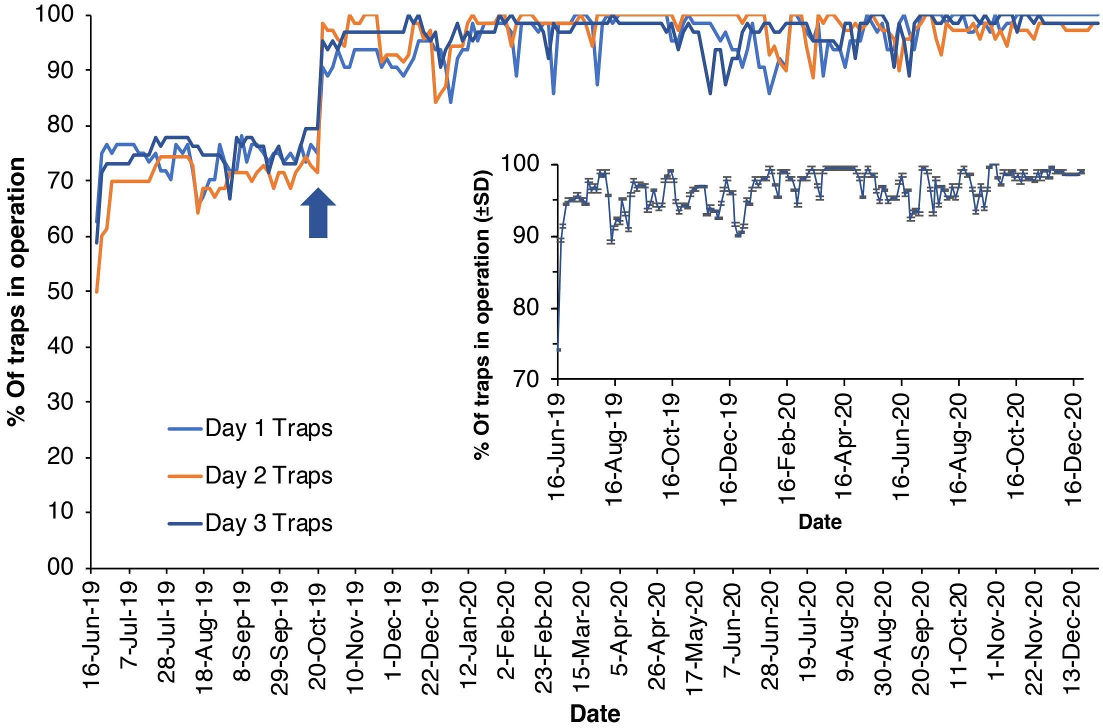

Figure S4. The percentage of Day 1, Day 2 and Day 3 traps in good working order at the time of servicing. The arrow indicates the time when an additional 45 BG-MosquitaireCO2 traps were deployed. The inset graph shows the 3-day average (±standard deviation) percentage of functional traps (overall 96,5 ± 2,9%). Day 1-3 trap locations are shown in Figure S3.

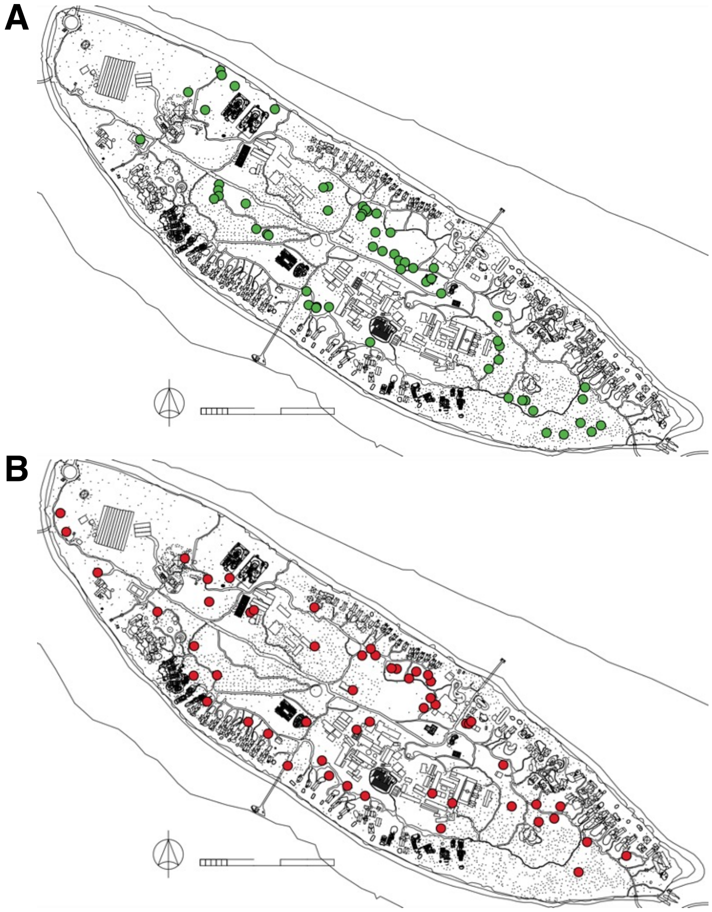

Figure S5. Fixed breeding sites targeted through larval source management (LSM). A: Locations of 56 tree ponds; B: Locations of 54 septic tanks.

A

1. (12/13 June 2019)
- Coconut shells: 2442
- Coconut boats: 869
- Open glass: 19
- Wine bottles: 12
- Plastic container: 11
- Clay pot: 1

2. (14-16 June 2019)
- Coconut shells: 30.640
- Coconut boats: 2375
- Wine bottles: 27
- Plastic container: 190

3. (17-18 June 2019)
- Coconut shells: 22.720
- Coconut boats: 3125
- Wine bottles: 300
- Plastic container: 600

4. (21 June 2019)
- Coconut shells: 10.638
- Coconut boats: 1700
- Wine bottles: 250

5. (23-24 June 2019)
- Coconut shells: 20.200
- Coconut boats: 3600
- Wine bottles: 20
- Plastic bottles: 216; Bags: 15

6. (24-27 June 2019)
- Coconut shells: 76.090
- Coconut boats: 15.815
- Glass bottles: 100
- Plastic bottles: 576
- Bags: 385

Total: 12-27 June 2019
- Coconut shells: 162.730
- Coconut boats: 27.484
- Glass bottles: 709
- Plastics: 1593

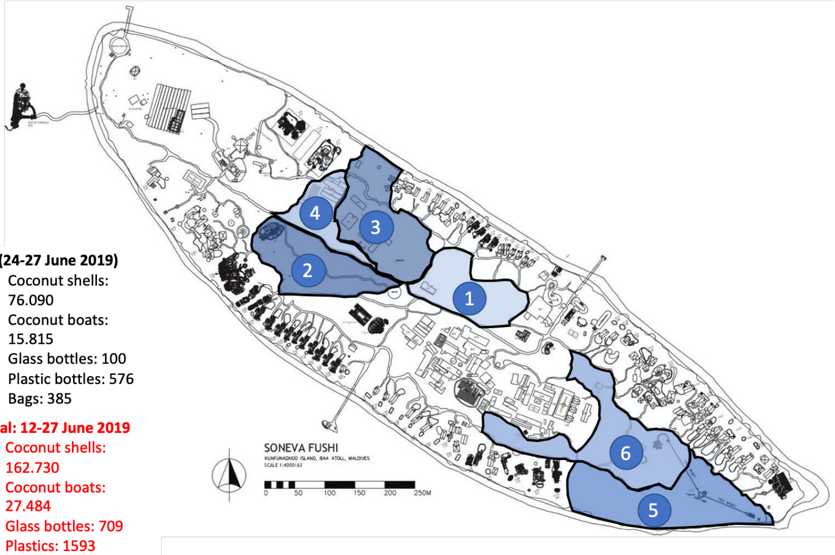

B

1. (26 June 2020)
- Coconut shells: 2800
- Coconut boats: 935

2. (27 June 2020)
- Coconut shells: 16.600
- Coconut boats: 8000

3. (28 June 2020)
- Coconut shells: 28.839
- Coconut boats: 2945

4. (29 June 2020)
- Coconut shells: 8.835
- Coconut boats: 3543

5. (30 June 2020)
- Coconut shells: 16.850
- Coconut boats: 8975

Total: 26-30 June 2020
- Coconut shells: 73.924
- Coconut boats: 24.398

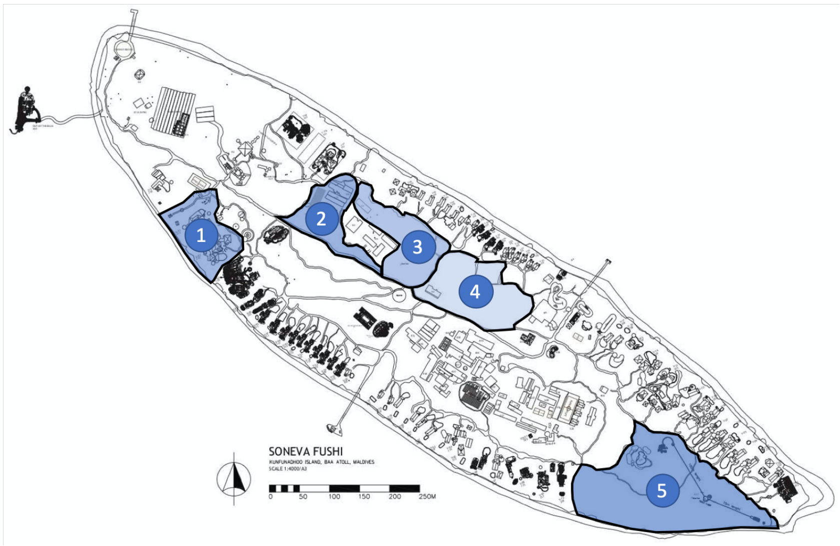

Figure S6. Removal of potential breeding sites from Kunfunadhoo island in 2019 (A) and 2020 (B). The most common breeding sites removed were empty coconut shells and coconut boats.

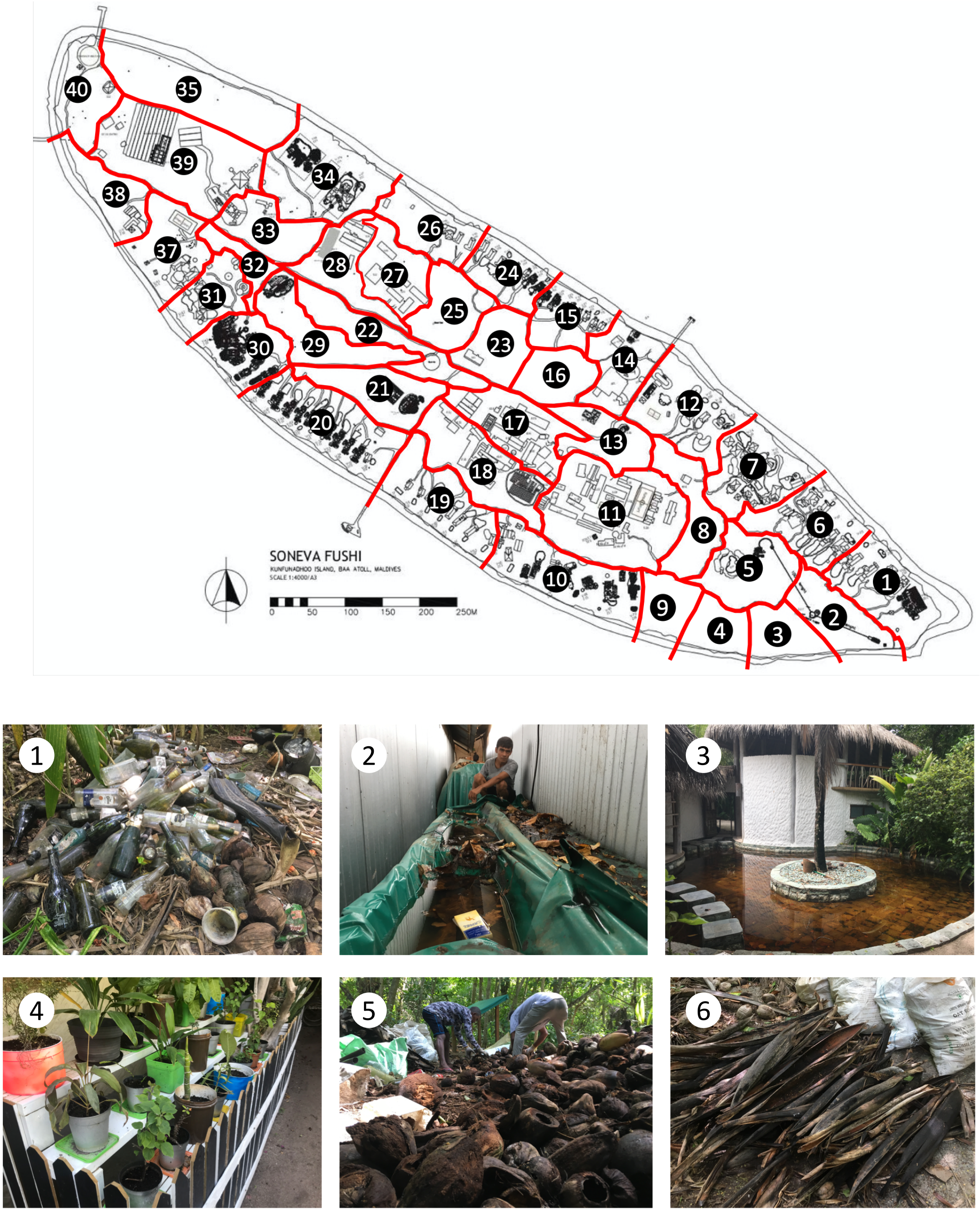

Figure S7. Division of Kunfunadhoo island into 40 sectors that are visited weekly to conduct larval source management (LSM). Examples of typical breeding sites removed were: 1) Trash that can hold water (e.g., bottles or tin cans), 2) tarpaulins used in construction, 3) temporarily abandoned ponds or pools, 4) saucers under flower/plant pots, 5) empty coconuts, 6) coconut boats.

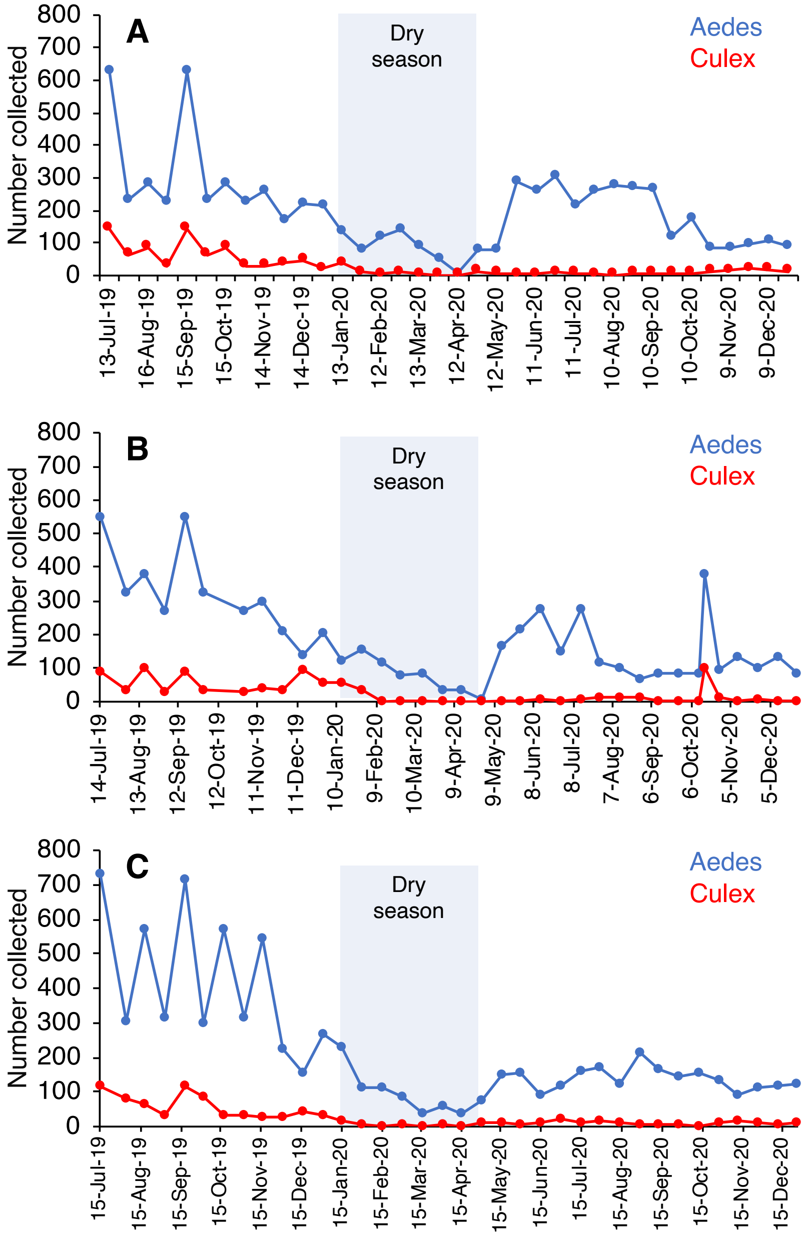

Figure S8. Absolute number of *Aedes albopictus* (in blue) and *Culex quinquefasciatus* (in red) collected from sticky cards of GAT traps over 2-week periods, July 2019-December 2020. A: catches from 100 GAT traps associated with the MosquitaireCO2 traps serviced on Day 1 (Fig. S3); B: similar, for 104 GAT traps associated with Day 2 traps; C: similar, for 100 GAT traps associated with Day 3 traps.

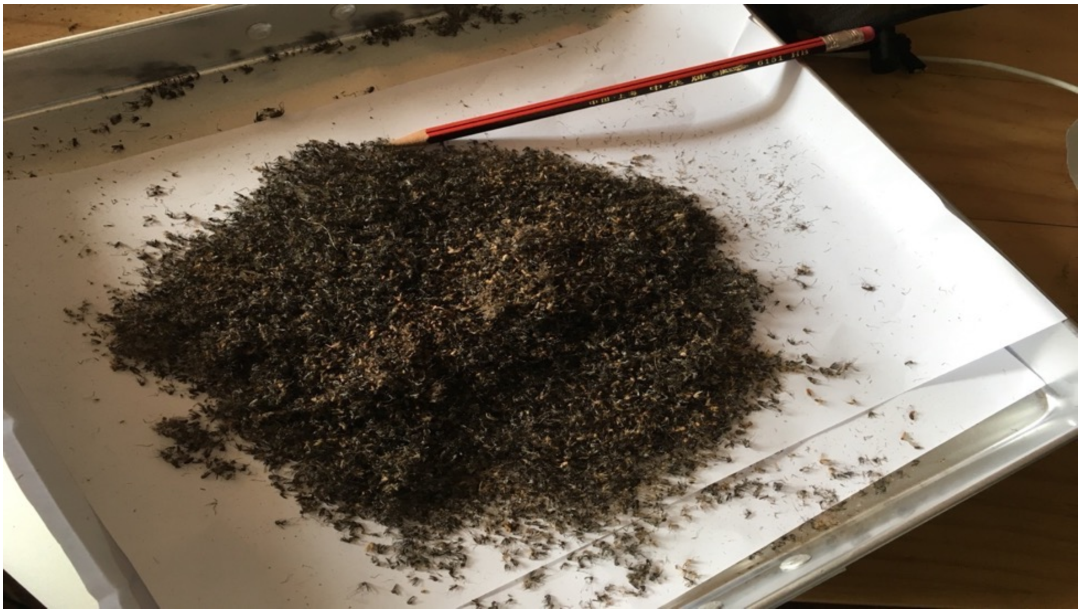

Figure S9. Mosquitoes collected from 150 BG-MosquitaireCO2 traps after the first month of operation. Between 16 June and 15 July 2019, a total of 113,085 Asian tiger mosquitoes (*Aedes albopictus*) and 14,559 Southern house mosquitoes (*Cx. quinquefasciatus*) were collected and individually counted. Over the entire 18-month trial period, 475,224 *Aedes* and 34,660 *Culex* mosquitoes were trapped.

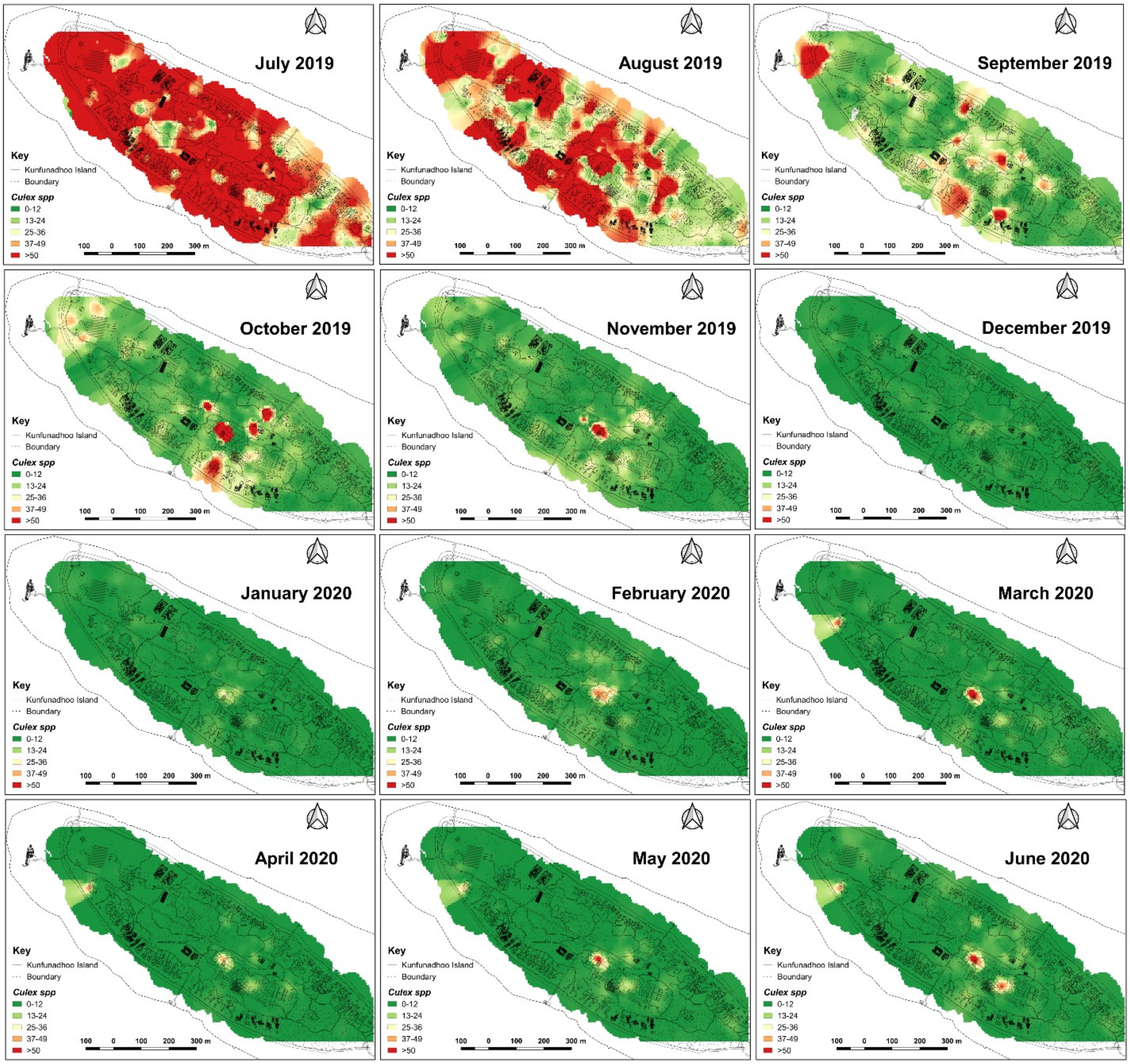

Figure S10. Inverse distance weighted heatmaps of *Culex quinquefasciatus* showing areas with high (red) or low (green) mosquito catches by month for the period June 2019 – June 2020. Impact of trapping was observed faster than for *Ae. albopictus* (Figure 7, main article) since *Culex* eggs cannot survive a period of drought.
